# Transdiaphragmatic Extension of a Pyogenic Liver Abscess Causing Purulent Pericarditis With Cardiac Tamponade in Septic Shock: A Case Report

**DOI:** 10.7759/cureus.106733

**Published:** 2026-04-09

**Authors:** Magdy Khames, Obaid Alzaabi, Saif Alkaabi, Abdalla Alnaqbi, Rady Ebead, Issam Marzouk, Mohamed Amin, Soliman Ahmed, Amit Gupta, Eisa Aldhaheri

**Affiliations:** 1 Intensive Care Unit, Zayed Military Hospital, Abu Dhabi, ARE; 2 Interventional Cardiology, Zayed Military Hospital, Abu Dhabi, ARE

**Keywords:** cardiac tamponade, eikenella corrodens, gastric sleeve complications, hepatic abscess, purulent pericarditis, septic shock, streptococcus anginosus, transdiaphragmatic spread

## Abstract

Purulent pericarditis is a rare, life-threatening condition in the post-antibiotic era, accounting for fewer than 1% of pericardial diseases, and may progress to cardiac tamponade. Transdiaphragmatic spread from a hepatic abscess is an exceedingly rare mechanism of infection. This case report highlights the unusual invasive potential of *Eikenella corrodens* in a polymicrobial infection that crossed anatomical barriers.

A 41-year-old man with a history of gastric sleeve surgery three years earlier presented with refractory septic shock and cardiac tamponade. Emergent ultrasound-guided pericardiocentesis with pericardial drain placement yielded 880 mL of purulent fluid. Concurrent CT imaging revealed a large hepatic abscess at the diaphragmatic dome with direct transdiaphragmatic continuity to the pericardium, highlighting a rare anatomical route of contiguous spread and providing a mechanistic explanation for the purulent pericardial infection. Cultures grew *Eikenella corrodens *and *Streptococcus anginosus. *Management included pericardial drainage, image-guided percutaneous drainage of the hepatic collections for source control, prolonged pathogen-directed antimicrobial therapy, and therapeutic anticoagulation for pulmonary embolism.

This case report describes a rare, life-threatening combination of septic shock, multiple pyogenic liver abscesses, purulent pericarditis with cardiac tamponade, and pulmonary embolism. Successful management required coordinated multidisciplinary care, including urgent pericardial drainage for source control and relief of hemodynamic compromise, prolonged pathogen-directed antimicrobial therapy guided by microbiology, and carefully timed anticoagulation for pulmonary embolism while balancing the competing risks of thrombosis and bleeding in the setting of concurrent cardiac tamponade.

## Introduction

Purulent pericarditis is an uncommon but life-threatening infection that requires rapid recognition, urgent drainage, and appropriate antimicrobial therapy. Although its incidence has declined in the antibiotic era, morbidity and mortality remain substantial, particularly when patients present with nonspecific symptoms, sepsis, or an unclear infectious source. Contemporary series still report high mortality despite treatment, commonly in the 20-30% range, and some reports cite rates up to ~40% depending on case mix and timing of diagnosis [1]. Historically, purulent pericarditis was frequently a complication of pneumonia, whereas modern cases more often occur in the context of healthcare exposure, thoracic surgery, immunosuppression, or bloodstream infection [2].

A pyogenic liver abscess is typically managed successfully with percutaneous drainage and targeted antibiotics; however, contiguous transdiaphragmatic extension into the thoracic cavity is very rarely described, and pericardial involvement progressing to cardiac tamponade has been reported only in isolated case reports [3]. The close anatomic relationship between the hepatic dome, diaphragm, pleura, and pericardium provides a potential pathway for direct spread, but only isolated reports describe progression to purulent pericarditis [4]. Polymicrobial infections involving abscess-forming organisms may further complicate the clinical course and antimicrobial selection, particularly when fastidious pathogens are involved [5,6].

This case describes a patient with a history of sleeve gastrectomy who developed septic shock from multiple pyogenic liver abscesses complicated by direct transdiaphragmatic extension to the pericardium, resulting in purulent pericarditis with cardiac tamponade caused by a polymicrobial infection with *Eikenella corrodens* and *Streptococcus anginosus*. It is presented to highlight the diagnostic value of early echocardiography and the importance of prompt multidisciplinary source control in an unusual clinical presentation.

## Case presentation

A 41-year-old male of Middle Eastern descent presented to the Emergency Department with a 3-day history of progressive substernal chest pain, dyspnea, and fever. His medical history was significant for a laparoscopic sleeve gastrectomy performed three years prior at another institution and recurrent perianal fistulas requiring multiple surgical interventions over the preceding two years. He had no known immunodeficiency, diabetes mellitus, or chronic steroid use. The patient was a non-smoker and had a history of chronic alcohol use that had ceased one year prior to presentation. This case report was prepared in accordance with the CARE guidelines [7]. Written informed consent was obtained from the patient for publication of this case report and accompanying images.

On initial assessment, the patient was diaphoretic, tachypneic, and appeared acutely distressed. His vital signs revealed hypotension, with a blood pressure of 78/44 mmHg, tachycardia at 126 beats per minute, a respiratory rate of 28 breaths per minute, a temperature of 39.2 °C, and oxygen saturation of 92% on room air. Cardiovascular examination revealed normal heart sounds that were relatively distant. Pulmonary examination revealed decreased breath sounds at the right base. The abdomen was soft, with mild right upper quadrant tenderness without guarding or rebound tenderness.

Initial laboratory investigations (Table 1) demonstrated marked leukocytosis with neutrophil predominance and markedly elevated inflammatory markers, consistent with severe sepsis. Evidence of organ dysfunction was also present, including acute kidney injury with elevated creatinine and blood urea nitrogen, mild transaminitis, and an elevated serum lactate of 4.8 mmol/L, indicating significant tissue hypoperfusion.

**Table 1:** Summary of laboratory investigations

Parameter	Result	Reference Range	Interpretation
White blood cells	24,500/μL	4,000-11,000/μL	Elevated
Neutrophilic	89%	40-75%	Elevated
Hemoglobin	11.2 g/dL	13.0-17.0 g/dL	Low
Platelet	198,000/μL	150,000-400,000/μL	Normal
C-reactive protein	312 mg/L	<5 mg/L	Elevated
Procalcitonin	48 ng/mL	<0.05 ng/mL	Markedly elevated
Creatinine	2.8 mg/dL	0.7–1.3 mg/dL	Elevated
Blood urea nitrogen (BUN)	42 mg/dL	7–20 mg/dL	Elevated
Aspartate aminotransferase (AST)	156 U/L	10–40 U/L	Elevated
Alanine transaminase (ALT)	198 U/L	7–56 U/L	Elevated
Lactate	4.8 mmol/L	0.5–2.0 mmol/L	Elevated

**Figure 1:**
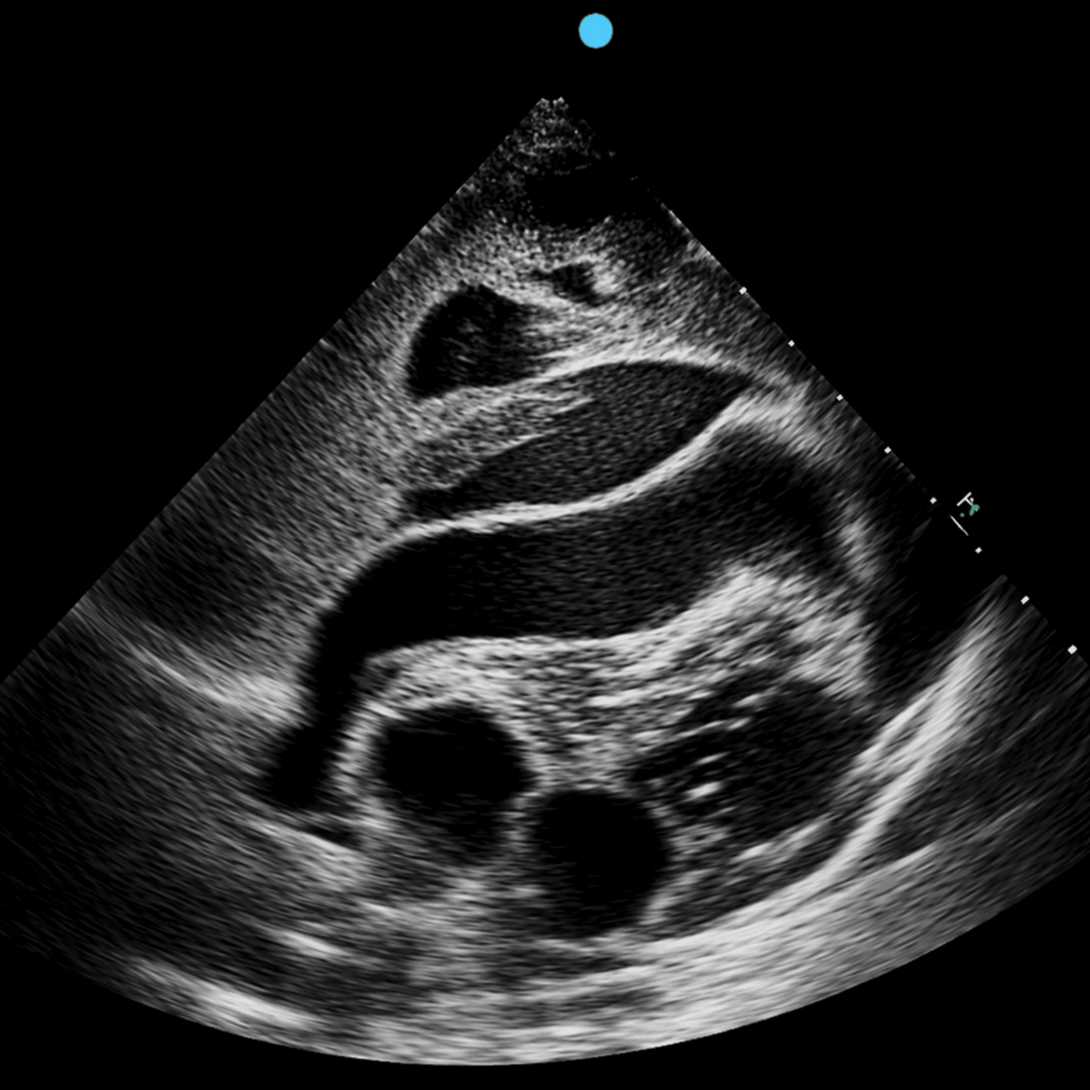
Initial POCUS showing pericardial effusion TTE demonstrated a large circumferential pericardial effusion with right ventricular diastolic collapse, consistent with cardiac tamponade. An additional atypical fluid collection was noted adjacent to the pericardium. POCUS: point-of-care ultrasound; TTE: transthoracic echocardiography

Initial assessment included point-of-care ultrasound (POCUS), which demonstrated a large circumferential pericardial effusion with right ventricular diastolic collapse, consistent with cardiac tamponade and significant hemodynamic compromise. An additional atypical fluid collection was noted adjacent to the pericardium, the significance of which was further evaluated on subsequent imaging (Figure 1).

**Figure 2:**
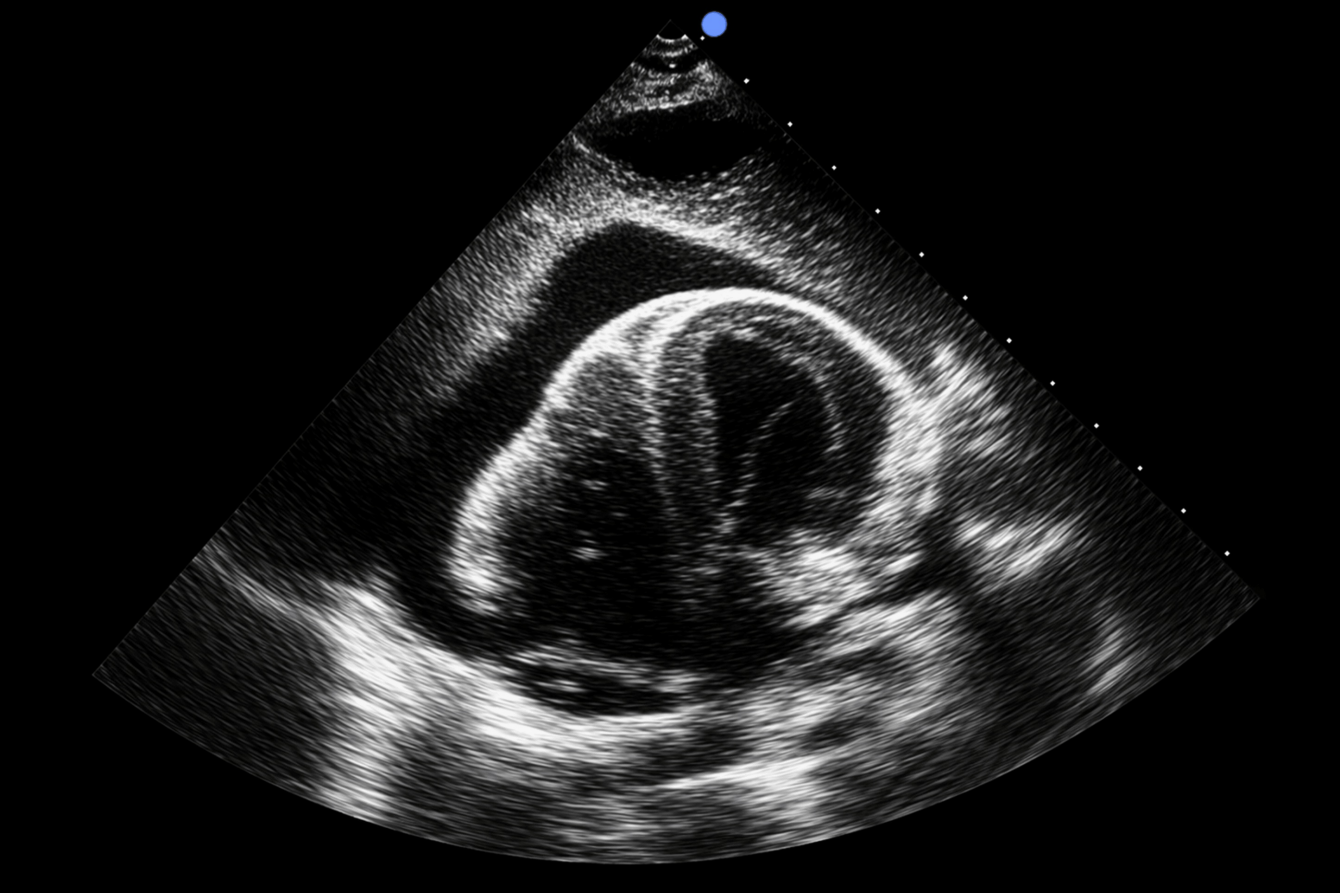
Additional echocardiography This view confirms a large pericardial effusion with tamponade physiology.

An additional transthoracic echocardiogram (TTE) confirmed a large pericardial effusion with echocardiographic features of cardiac tamponade and hemodynamic compromise (Figure 2).

**Figure 3:**
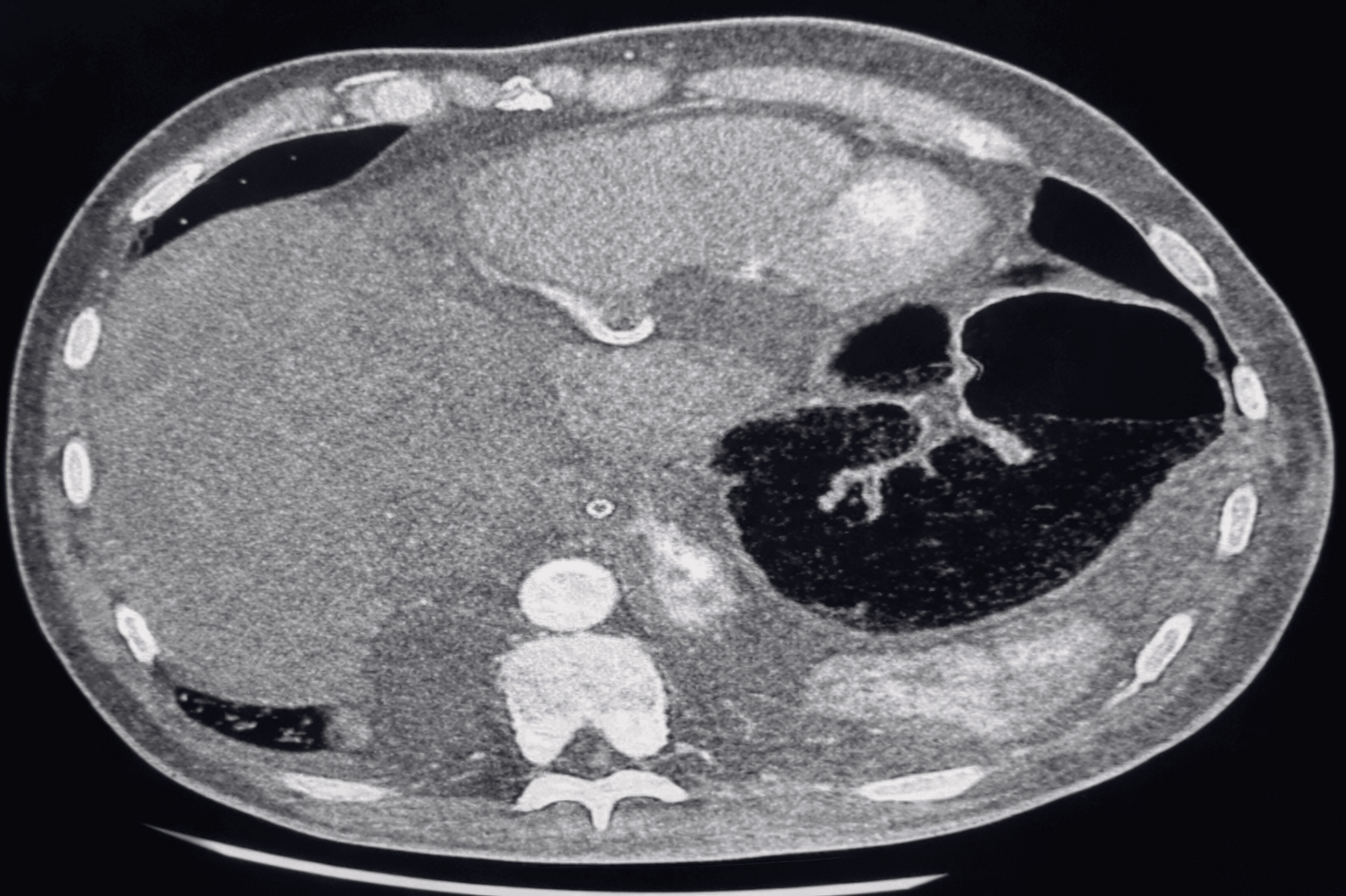
Abdominal contrast-enhanced CT scan Contrast-enhanced CT scan demonstrating a 9.2 cm hepatic abscess in segment VIII at the diaphragmatic dome.

Contrast-enhanced computed tomography (CECT) of the chest and abdomen revealed a 9.2 cm hepatic abscess in segment VIII involving the diaphragmatic surface, with direct inflammatory extension into the pericardial space (Figure 3). There was evidence of diaphragmatic thickening and discontinuity at the region of contact with the hepatic dome, suggesting transdiaphragmatic spread. In addition, pulmonary embolism was identified on Chest CT.

Microbiological analysis of pericardial and hepatic aspirates identified *Eikenella corrodens* and *Streptococcus anginosus* (Milleri group). Blood cultures obtained prior to antibiotic administration also grew both organisms, with the antimicrobial susceptibility profile shown in Table 2. This combination was clinically significant, as it supported a polymicrobial invasive process, with *Eikenella corrodens* representing an unusual pathogen in a deep hepatopericardial infection.

**Table 2:** Antimicrobial susceptibility profile

Antibiotics	Streptococcus anginosus	Eikenella corrodens
Amoxicillin/Clavulanate	Not tested	Susceptible
Ampicillin	Not tested	Susceptible
Benzylpenicillin	Susceptible	Not tested
Ceftriaxone	Susceptible	Susceptible
Ciprofloxacin	Not tested	Resistant
Tetracycline	Not tested	Susceptible
Vancomycin	Susceptible	Not tested

A standardized sepsis bundle was implemented immediately upon recognition of septic shock. The patient received broad-spectrum antibiotics within one hour of presentation: vancomycin 1.5g IV and piperacillin-tazobactam 4.5g IV, along with 2L of crystalloid fluid resuscitation. Emergent pericardiocentesis was performed under ultrasound guidance using a subxiphoid approach, draining 880mL of frank pus initially; the total drained volume was 1000mL. An 8 French pigtail catheter was left in place for continued drainage.

Despite initial pericardial drainage, antimicrobial therapy, and hemodynamic resuscitation, the patient initially deteriorated, requiring escalating vasopressor support with norepinephrine up to 0.8 mcg/kg/min and vasopressin at 0.04 units/min. He subsequently developed oliguric acute kidney injury with fluid overload, prompting the initiation of continuous renal replacement therapy (CRRT). Clinical improvement followed ongoing source control and antimicrobial treatment, with gradual hemodynamic stabilization thereafter. CT-guided percutaneous drainage of the hepatic abscess was performed on day 2, with placement of a 12 French drainage catheter, which yielded an additional 120 mL of purulent material. In view of the pulmonary embolism and the temporary inability to initiate anticoagulation, an inferior vena cava filter was inserted by interventional radiology until therapeutic anticoagulation could be safely started.

Following microbiological identification of the organisms on day 3, antibiotic therapy was de-escalated to ceftriaxone 2g IV daily and metronidazole 500mg IV every 8 hours. The patient completed four weeks of intravenous therapy, followed by four weeks of oral amoxicillin-clavulanate.

**Figure 4:**
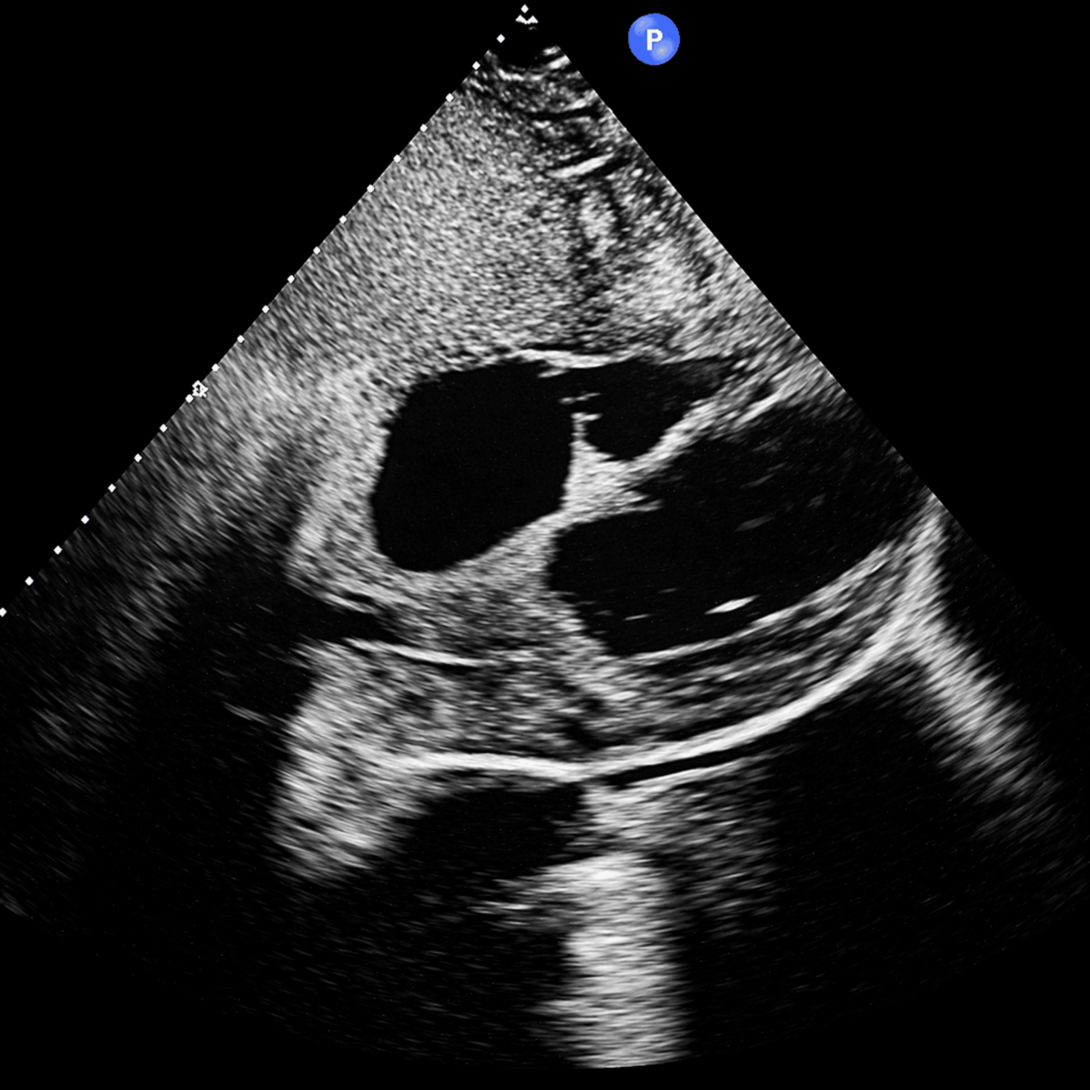
The follow-up echocardiography Transthoracic echocardiography demonstrated normal cardiac function with no pericardial effusion.

The patient's clinical course improved gradually over the following two weeks. Vasopressors were successfully weaned by day 5, and CRRT was discontinued by day 8 with recovery of renal function. The pericardial drain was removed on day 7 after output decreased to less than 30 mL/day, and therapeutic anticoagulation was initiated thereafter. The hepatic drain was removed on day 14 after serial imaging demonstrated collapse of the abscess cavity. Transthoracic echocardiography (TTE) demonstrated normal cardiac function with an ejection fraction of 60%, no pericardial effusion, and no evidence of constrictive physiology (Figure 4).

The patient was discharged home, with outpatient follow-up planned at three months. Repeat CT imaging will be performed at that time to assess the resolution of the hepatic and pericardial collections and to evaluate for any residual.

**Figure 5:**
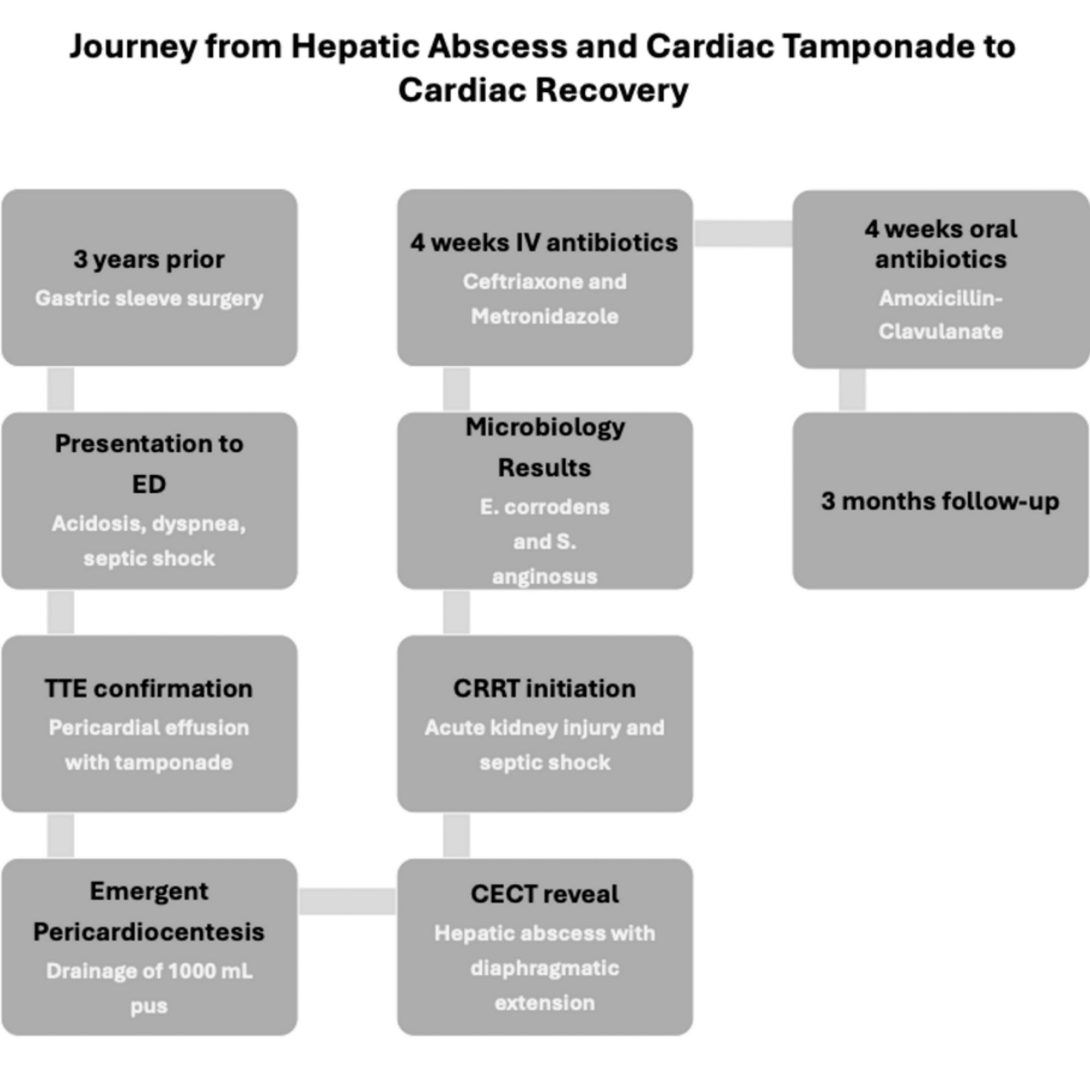
Timeline Timeline showing the journey from initial presentation to complete cardiac recovery. Generated using Microsoft PowerPoint (Microsoft Corp., Redmond, WA, US) ED: emergency department; TTE: transthoracic echocardiography; CECT: contrast-enhanced computed tomography; CRRT: continuous renal replacement therapy*; E. corrodens: Eikenella corrodens; S. anginosus: Streptococcus anginosus*

Figure 5 summarizes the patient’s entire clinical course, from initial presentation and diagnostic workup through source-control interventions, antimicrobial therapy, and planned follow-up.

## Discussion

This case represents an extraordinary example of transdiaphragmatic spread of infection from a hepatic source to the pericardium, resulting in life-threatening cardiac tamponade. A possible explanation is that the invasive nature of *Eikenella* *corrodens*, together with the patient’s history of gastric surgery, may have contributed to this pattern of spread, although the precise mechanism remains speculative [8]. The presence of *Streptococcus anginosus *as a co-pathogen was also notable, given its recognized association with abscess formation in polymicrobial infections.

*Eikenella corrodens* is frequently recovered in polymicrobial infections with streptococci, and experimental data suggest that mixed-species interactions, including coaggregation and oxygen consumption by companion organisms, may facilitate Eikenella growth and persistence in deep-seated abscesses [9]. Earlier case literature also supports the invasive potential of *Eikenella corrodens*, including purulent pericarditis with mediastinal extension, reinforcing its ability to cause severe deep-compartment infection [10]. A 2019 case report described severe purulent pericarditis caused by invasive *Eikenella corrodens* in an immunosuppressed individual, emphasizing the organism's pathogenic potential [11].

The *Streptococcus anginosus* group is an important pathogen in pyogenic liver abscesses and is well-recognized for its propensity to cause abscess formation and invasive disseminated infection. A 2022 review highlighted the clinical relevance of the group across deep-seated infections and emphasized its pyogenic behavior and diagnostic importance [6]. Purulent pericarditis usually arises from contiguous intrathoracic infection, hematogenous spread, or postoperative infection, whereas hepatic-source disease is exceptionally uncommon and reported mainly as isolated case reports [12,13]. The role of prior sleeve gastrectomy in this case merits consideration. A pyogenic hepatic abscess is a rare complication reported after laparoscopic sleeve gastrectomy, most often described in isolated case reports and small case series rather than large incidence studies. Proposed mechanisms include staple-line leak or infected postoperative collections with secondary bacterial seeding, and prior surgery may alter local anatomy and tissue planes, potentially facilitating a contiguous spread of infection [14-16].

The use of CRRT helped manage the metabolic derangements of severe sepsis and supported fluid balance during the acute phase of tamponade recovery. Current evidence supports early initiation of renal replacement therapy in septic patients with acute kidney injury, particularly those with fluid overload refractory to medical management [17]. The primary strength of this report is the definitive microbiological identification and clear radiological evidence of the transmission route. A limitation is the lack of specific gastric imaging to rule out a subtle marginal ulcer perforation as the sentinel event for the hepatic abscess.

## Conclusions

This case highlights a rare, life-threatening constellation of septic shock due to multiple pyogenic liver abscesses complicated by purulent pericarditis with cardiac tamponade and pulmonary embolism. The patient’s favorable outcome appeared to be associated with rapid recognition of pericardial involvement, urgent drainage for source control, and coordinated multidisciplinary management. Targeted, prolonged antimicrobial therapy guided by microbiological identification likely contributed to control of the deep-seated infection, while anticoagulation required careful timing to balance thrombotic risk against procedural and bleeding concerns. This case highlights that severe intra-abdominal sepsis may, in some instances, be associated with occult cardiac complications, with early echocardiography proving clinically useful when hemodynamic instability appears disproportionate or unexplained. Prompt imaging, timely interventions, and close follow-up may contribute to recovery in such complex presentations.
